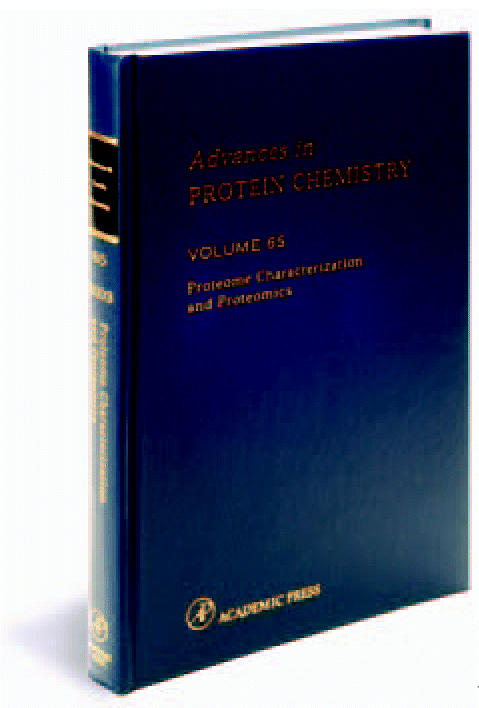# Advances in Protein Chemistry, Volume 65: Proteome Characterization and Proteomics

**Published:** 2004-08

**Authors:** Barbara A. Wetmore, B. Alex Merrick

**Affiliations:** Barbara A. Wetmore is a senior postdoctoral fellow in the Proteomics Group at NIEHS. Her research involves site-specific phosphorylation of p53 in cancer research. B. Alex Merrick heads the Proteomics Group in the National Center for Toxicogenomics with the mission of identifying key proteins and pathways involved in toxicant exposure.

Edited by Richard D. Smith and Timothy D. Veenstra

San Diego, CA:Academic Press, 2003. 413 pp. ISBN: 0-12-034265-0, $139.95 cloth

Proteins, like characters in a novel, can be described by their appearance, behavior, interactions, peculiarities in specific situations, and movement in time and space. Also like characters in a novel, proteins are created and undergo maturation but eventually cease to function and are eliminated. By analogy, proteomics has the formidable task of describing extremely large sets of proteins, or “proteomes,” within organisms. *Proteome Characterization and Proteomics* delivers an excellent balance of state-of-the-art technologies, chemistries, and instrumentation designed to measure proteomes and is supplemented by applications of proteomics to biologic problems in species ranging from single-cell to complex organisms.

The beginning chapter, “Proteomics in the Postgenomic Age,” traces completion of the human genome project to the development of transcriptomics and proteomics. The authors highlight the comparative higher complexity of functioning proteins, the difficulty of predicting post-translational processing from mRNA sequence alone, and the frequent disparity between mRNA levels and protein expression, suggesting that only direct analysis of the proteome itself can characterize proteins with certainty. “The Tools of Proteomics” lucidly explains the types of mass spectrometers and ionization methods available and describes their use and impact in proteomics. Subsequent chapters describe long-standing protein separation techniques including two-dimensional (2D)-gel electrophoresis, liquid chromatography (LC), and capillary electrophoresis.

A recent instrumental refinement for greater sensitivity is discussed in a chapter on the use of accurate mass tags generated during Fourier transform ion cyclotron resonance mass spectrometry (FTICR MS) to determine protein identity. FTICR MS, assisted by the DREAMS algorithm (dynamic range enhancement applied to mass spectrometry) makes possible very high mass measurement accuracy (MMA). The following chapter provides an excellent discussion of quantitative proteomic techniques, covering 2D-PAGE techniques, multiplexing, metabolic and postextraction labeling, and isolation and quantitation of phosphopeptides. A chapter covering post-translational modifications describes detection of phosphorylated and glycosylated proteins as well as immunoaffinity chromatography, phosphopeptide mapping, and isotopic labeling and collision-induced dissociation strategies. An approach to mapping post-translational modifications at the amino acid level using LC-MS-MS is nicely described in a section discussing a scoring algorithm for spectra analysis (SALSA).

Advances in structural and functional proteomics are described in a chapter where electrospray ionization mass spectrometry is showcased in studies of noncovalent protein complexes. In a section devoted to proteomic strategies in drug discovery, the authors stress the need for higher-throughput, parallel-analysis platforms to fulfill the pharmaceutical industry’s pressing needs for mass screening. The final section discusses proteomics and bioinformatics, reviewing the DNA and protein sequence databases, 2D-gel annotated databases, and the impending need for genome and proteome database integration.

Topics that the authors do not explore are the explosive growth in proteomics in disease diagnosis, biomarker discovery, and drug-toxicant profiling using retentate chromatography mass spectrometry (RC-MS), and also the growing number of protein/antibody arrays. In one of the most significant and controversial success stories of proteomics, the use of RC-MS has already given cancer researchers a proteomic serum signature that can detect ovarian cancer at an earlier stage than previously possible. Further, microarrays of antibodies or other affinity ligands hold great promise for large parallel analysis at an economy of sample volume and expense.

Overall, this is a well-written volume on the current state of proteomics technologies. The editors convey an understanding of the latest developments in mass spectrometry, protein fractionation and applications. This volume is essential for use of proteomic tools in global protein characterization and discovery research. Like any good novel, researchers are finding that each proteome has a cast of thousands of proteins requiring careful study, characterization, and a means for sophisticated interpretation.

## Figures and Tables

**Figure:**